# *In vitro* activity of the first-in-class triazaacenaphthylene gepotidacin alone and in combination with doxycycline against drug-resistant and -susceptible *Mycoplasma genitalium*

**DOI:** 10.1080/22221751.2020.1775498

**Published:** 2020-06-18

**Authors:** Jørgen Skov Jensen, Christina Nørgaard, Nicole Scangarella-Oman, Magnus Unemo

**Affiliations:** a Department of Bacteria, Parasites and Fungi, Research Unit for Reproductive Tract Microbiology, Statens Serum Institut, Copenhagen, Denmark; b GlaxoSmithKline, Collegeville, Pennsylvania, USA; c Örebro University, Örebro, Sweden

**Keywords:** Mycoplasma genitalium, antimicrobial resistance, gepotidacin, in-vitro susceptibility testing, combination therapy

## Abstract

*Mycoplasma genitalium* has developed resistance to first-line azithromycin and second-line moxifloxacin. Third-line pristinamycin is only 75% effective. Gepotidacin, a novel triazaacenaphthylene topoisomerase II inhibitor, blocks bacterial DNA replication. We determined the *in vitro* activity of gepotidacin alone and in combination with doxycycline against a diverse collection of *Mycoplasma genitalium* isolates (*n* = 54).

Minimum inhibitory concentrations (MICs) and minimum bactericidal concentrations (MBCs) were determined by a Vero-cell culture method. Macrolide resistance was present in 31 (57%) isolates, fluoroquinolone resistance in 18 (33%) isolates, and 17 (31%) had dual resistance. Synergy testing was performed for gepotidacin and doxycycline by checkerboard analysis for two macrolide- and two dual-resistant isolates.

Gepotidacin was active against all 54 *M. genitalium* isolates with median and modal MICs of 0.125 mg/L and MIC_90_ of 0.25 mg/L (range ≤0.016–0.5 mg/L). No difference in gepotidacin MIC between macrolide-resistant and -susceptible isolates (*p* = 0.24) or between fluoroquinolone-, dual-resistant and -susceptible isolates (*p* = 0.2) was demonstrated. Gepotidacin MBCs were available for 44 *M. genitalium* isolates with median MIC of 0.064 mg/L and median MBC of 0.125 mg/L. All isolates had ≤4-fold difference between MIC and MBC, suggesting bactericidal effect for gepotidacin. Checkerboard analysis indicated synergistic effect for gepotidacin in combination with doxycycline [fractional inhibitory concentration index (ΣFICI) of 0.5] for two isolates and additive/indifference (ΣFICI at 0.62 and 0.75) for two isolates.

Gepotidacin warrants further evaluation in clinical treatment trials for *M. genitalium.* Combination therapy with doxycycline should be clinically studied to assess effect and potential protection against development and/or spread of gepotidacin resistance.

## Introduction

*Mycoplasma genitalium* is an emerging cause of sexually transmitted infections (STIs) accounting for approximately 25% of non-chlamydial-non-gonococcal urethritis, 10%–15% of cervicitis and 10%–15% of pelvic inflammatory disease [1,2]. While globally, gonorrhoea is considered to be the second most prevalent bacterial STI [3], *M. genitalium* has been reported as the second most common bacterial STI after *Chlamydia trachomatis* in some studies [4,5].

First-line treatment is azithromycin [6], but rates of resistance are increasing globally and are currently exceeding 40% in most regions [7]. Macrolide resistance is caused by single-base mutations in positions A2058 or A2959 (*Escherichia coli* numbering) in region V of the 23S rRNA [8], which is present in only one copy in the genome. Thus, one mutational event can change the susceptibility phenotype from extremely susceptible to highly resistant. Moxifloxacin is the second-line therapy [6] but mutations in the quinolone-resistance-determining-region (QRDR) of ParC (primarily amino acid positions S83 and D87; *M. genitalium* numbering) have been increasingly reported with rates below 10% in most European countries [9,10] to 30%–40% in some populations in North America [11] and as high as 88% (dual resistance >80%) in China [12]. With dual resistance to macrolides and fluoroquinolones, treatment options are extremely limited. Third-line pristinamycin [6] is difficult to source and only 75% effective [13]. Consequently, there is an urgent need for alternative therapies for which there is no cross-resistance, and for strategies to protect new antimicrobials against rapid development and/or spread of resistance. Accordingly, combination therapy and resistance-guided sequential therapy [14] are important to consider.

Gepotidacin is a novel, first-in-class triazaacenaphthylene topoisomerase II inhibitor which blocks bacterial DNA replication [15]. As with fluoroquinolones, GyrA and ParC are targeted by gepotidacin, but the target sequences and the mechanism of action of gepotidacin are distinct from the fluoroquinolones [15]. One prior study has shown gepotidacin *in vitro* activity against a small number (*n* = 10) of fluoroquinolone- and macrolide-susceptible *M. genitalium* isolates [16]. With the increasing levels of fluoroquinolone resistance, we considered it essential to test a larger collection of isolates, including many that are fluoroquinolone- and macrolide-resistant and to study also interactions with doxycycline, which has become recommended for pre-treatment before resistance-guided sequential therapy [17,18].

## Methods

### *M. genitalium* isolates

A collection of 54 *M. genitalium* isolates originating from 51 patients were tested (Table 1). These included the *M. genitalium* G37 type-strain, an early passage of the M30 strain isolated by David Taylor-Robinson in 1980 [19] and obtained from the Mollicutes Collection (Gainesville, FL, USA). One additional isolate was kindly provided by Pat Totten, Seattle, USA. The remaining 51 isolates were cultured in Copenhagen from samples collected in seven countries from 1996 to 2016. Thirty-one (57%) isolates were macrolide-resistant with azithromycin MICs of ≥16 mg/L and mutations in 23S rRNA gene positions A2058 (*n* = 17) or A2059 (*n* = 14). Eighteen (33%) isolates were moxifloxacin-resistant (MIC ≥1 mg/L), with ParC amino acid alterations in position S83 (*n* = 14 [12 S83I, 2 S83R]) and D87 (*n* = 4 [3 D87N, 1 D87Y]). Finally, 17 (31%) isolates had dual-class resistance and were resistant to both moxifloxacin and azithromycin (Table 1).

**Table 1.** Distribution of 54 *Mycoplasma genitalium* isolates according to country of origin and antimicrobial resistance.

Country of origin	No. of isolates	No. of macrolide-resistant isolates	No. of fluoroquinolone-resistant isolates	No. of dual-class resistant isolates
Sweden	15	6	2	2
Australia	13	12	6	6
Denmark	9	4	3	3
Norway	7	7	5	5
France	3	0	0	0
Japan	3	0	1	0
UK	3	1	1	1
USA	1	1	0	0

No., number.

### Determination of minimum inhibitory concentration (MIC)

Gepotidacin, azithromycin, moxifloxacin, and doxycycline MICs were determined by inoculating approximately 5000 genome equivalents (geq) as determined by a quantitative PCR (qPCR) assay [20] into a Vero-cell culture containing two-fold dilutions of the test-antimicrobial compound [21]. After a three-week incubation period, cells and supernatant were harvested and growth of *M. genitalium* was determined by the same qPCR assay. MIC was defined as the minimal concentration of the test-antimicrobial causing a 99% inhibition of growth when compared to the mean of the control cultures grown without antimicrobial.

### Determination of minimum bactericidal concentration (MBC)

After incubation for MIC determination, 15 µL of culture medium was transferred from the MIC plate to 135 µL fresh Vero-cell suspension, resulting in a 10-fold dilution of gepotidacin. Plates were sealed and incubated for four weeks and *M. genitalium* growth was subsequently determined by qPCR. MBC was defined as the minimal concentration of the test-antimicrobial causing a 99% inhibition of growth when compared to the mean of the control culture wells grown without antimicrobial.

### Synergy testing for gepotidacin combined with doxycycline

Checkerboard analysis representing 8 by 8 two-fold dilutions of gepotidacin and doxycycline, with the mid-point concentration representing the gepotidacin and doxycycline MICs, respectively, were prepared in Vero-cell suspensions and *M. genitalium* inoculum was incubated for three weeks before growth was determined by a qPCR assay [20]. MICs were defined as described above. Results were described as the fractional inhibitory concentration index (FICI) [22]. Synergy was assumed when FICI ≤ 0.5, antagonism when FICI >4 [23].

### Statistical methods

Statistical analysis of the MIC data was performed using StatsDirect version 3.1. For pairwise comparisons between groups, the Mann–Whitney test was used. For comparisons across different antibiotics, the Kruskal–Wallis test was applied. For multiple comparisons, the results obtained by the Dwass-Steel-Chritchlow-Fligner method were used.

## Results

**Figure 1.**
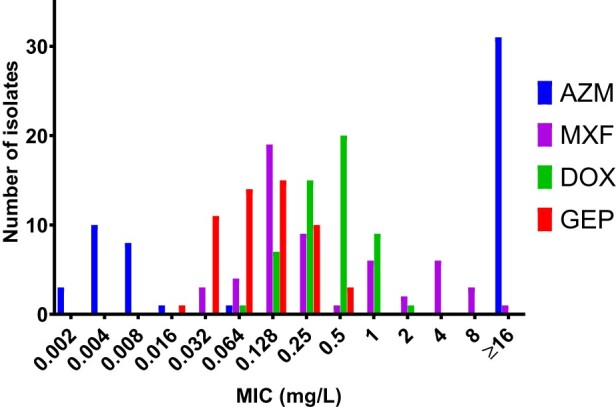
Distribution of gepotidacin (GEP), azithromycin (AZM), moxifloxacin (MXF), and doxycycline (DOX) MICs for 54 *Mycoplasma genitalium* isolates as determined by a Vero-cell culture-based assay [21].

Gepotidacin was active *in vitro* against all 54 *M. genitalium* isolates tested with a median and a modal MIC of 0.125 mg/L and a MIC_90_ of 0.25 mg/L (range ≤0.016–0.5 mg/L) (Figure 1). No difference in gepotidacin MICs between macrolide-resistant and -susceptible isolates (*p* = 0.24) or between fluoroquinolone-, dual-class-resistant or -susceptible isolates (*p* = 0.2) was observed.

In this selected collection of isolates, gepotidacin was the most active compound tested (Table 2) with an MIC_90_ of 0.25 mg/L compared with azithromycin (MIC_90_ of >64 mg/L), moxifloxacin (MIC_90_ of 4 mg/L), and doxycycline (MIC_90_ of 1 mg/L) (*p* < 0.001 for all comparisons)

**Table 2.** MICs of 36 moxifloxacin-susceptible and 18 moxifloxacin-resistant isolates of *Mycoplasma genitalium*.

Antibiotic	MIC_50_ [mg/L]	MIC_90_ [mg/L]	MIC range [mg/L]
Gepotidacin	0.125	0.25	≤0.016-0.5
Gepotidacin (moxifloxacin susceptible)	0.125	0.25	0.032-0.5
Gepotidacin (moxifloxacin resistant)	0.064	0.25	≤0.016-0.5
Azithromycin	16	>64	0.002->64
Doxycycline	0.5	1	0.06-2
Moxifloxacin	0.25	4	0.03->16

Gepotidacin MBCs were available for 44 *M. genitalium* isolates and demonstrated a median MIC of 0.064 mg/L and a median MBC of 0.125 mg/L. All isolates had a ≤4-fold difference between MIC and MBC suggesting a bactericidal effect for gepotidacin. Ten isolates failed MBC testing mostly due to failure to reach the required growth in the growth controls.

Checkerboard analysis of gepotidacin combined with doxycycline indicated a synergistic or additive/indifferent effect with a FICI of 0.5 for two isolates (one macrolide-resistant and one dual-resistant) and a FICI of 0.62 and 0.75 for a macrolide- and a dual-resistant isolate, respectively (Details given in Table 3). Most importantly, no antagonistic interactions were identified.

**Table 3.** Fractional inhibitory concentration index (FICI) and MIC (mg/L) of selected antimicrobials for four *Mycoplasma genitalium* isolates. Synergy is defined as FICI ≤0.5 and antagonism as FICI >4 [23]. Macrolide resistance mediating mutations in the 23S rRNA gene according to *Escherichia coli* numbering. ParC mutations leading to moxifloxacin resistance according to *M. genitalium* numbering.

	FICI DOX/GEP	23S rRNA gene mutation	ParC mutation	GyrA mutation	DOX	AZM	MXF	GEP
M6257	0.5	A2058G	WT	WT	0.5	>64	0.25	0.032
M6320	0.62	A2059G	WT	WT	0.25	≥64	0.063	0.125
M6926	0.75	A2058T	S83I	WT	0.5	16	4	0.064
M6984	0.5	A2058G	D87N	WT	0.5	>64	1	0.125

DOX: Doxycycline, AZM: Azithromycin, MXF: Moxifloxacin, GEP: Gepotidacin.

## Discussion

Gepotidacin inhibited all *M. genitalium* isolates at MICs ≤0.5 mg/L and was significantly more active than the comparator antibiotics against the *M. genitalium* isolates tested. These included geographically, temporally and genetically diverse reference strains and clinical *M. genitalium* isolates with a high proportion of fluoroquinolone- (33%) and macrolide-resistant (57%) isolates. No evidence of cross-resistance to the tested comparators was observed, and most importantly, no difference in gepotidacin MIC was seen between isolates resistant or susceptible to the fluoroquinolone moxifloxacin (also a topoisomerase II inhibitor targeting ParC and GyrA). Gepotidacin demonstrated bactericidal activity and a synergistic interaction in combination with doxycycline was identified in two of the four isolates tested, with an additive/indifferent effect in the remaining two isolates. Most importantly, no antagonistic interaction between gepotidacin and doxycycline was observed.

The main strength of the present study is the unique *M. genitalium* isolate collection representing a wide range of resistance-mediating mutations, but a potential limitation is the use of the Vero-cell culture-based assay, which is not internationally validated and approved. However, at present, no internationally validated, quality assured and approved methods or guidelines for antimicrobial susceptibility testing of *M. genitalium* exist although the CLSI standard for antimicrobial susceptibility testing of *M. pneumoniae* [24] has been used for *M. genitalium* isolates capable of axenic growth (cell-free, in artificial media) [16]. In that study [16], the broth dilution method resulted in gepotidacin MIC values that were significantly lower than those reported in the present study using the Vero-cell based assay. They found a gepotidacin MIC_90_ at 0.032 µg/mL for 10 macrolide- and moxifloxacin-susceptible isolates, which was somewhat lower than that found among the 25 isolates of the closely related *M. pneumoniae* isolates tested which had a gepotidacin MIC_90_ at 0.125 mg/L, more in line with the findings from this current study. In contrast, although the CLSI method deemed gepotidacin bacteriostatic with three or more dilution steps between MIC and MBC, the cell-based assay had very similar MIC and MBC values documenting bactericidal activity. This finding is more in line with findings for gepotidacin from other Gram-positive and Gram-negative bacteria [25] and is to be expected from gepotidacin’s mechanism of action, which in many ways is similar to that of the bactericidal fluoroquinolones. It is well described that the Vero-cell culture-based method may report higher MICs for some antimicrobials such as erythromycin as compared to the broth dilution assay [21], but the clinical relevance of this finding is unclear. Furthermore, the Vero-cell culture-based method is essential when a large and representative isolate collection has to be examined, as many newer clinical strains are incapable of axenic growth even despite extensive attempts to *in vitro* adaptation.

It was encouraging that even dual-class resistant isolates with high MICs for azithromycin and moxifloxacin had a gepotidacin MIC_90_ of 0.25 mg/L with the highest gepotidacin MIC at 0.5 mg/L. In a recent phase II randomized controlled clinical trial (RCT) of single-dose gepotidacin therapy for gonorrhoea, the three urogenital treatment failures all had pre-treatment gepotidacin MICs of 1 mg/L [26]. Whether a multi-dose regimen, which will be needed for *M. genitalium*, would have a similar clinical resistance breakpoint is unclear.

The synergy study showed a general trend toward synergy or at least an additive effect when combining gepotidacin and doxycycline. Although only half of the examined isolates (*n* = 4) showed a FICI ≤ 0.5 suggestive of a synergy, the remaining two isolates showed FICI at 0.62 and 0.75, respectively, not sufficiently low enough to be considered synergistic [23], but still clearly showing lack of antagonism. This is important, as tetracyclines are the only antimicrobials where clear-cut *in vitro* resistance has not been demonstrated. Despite low cure rates of 30%–40% [27], doxycycline has been shown to significantly decrease the *M. genitalium* organism load during treatment and to decrease the selection of macrolide resistance during resistance-guided sequential therapy [14]. In its original concept, this approach uses 7 days of doxycycline before changing to macrolide or fluoroquinolone therapy according to results of molecular macrolide resistance testing [14]. However, this means 14 days of antimicrobial therapy which increases the selection of antimicrobial resistance in other bacteria and which may lead to decreased patient compliance. Future studies should take advantage of potential synergistic or additive effects and limit the pre-treatment to a few days before continuing with dual therapy. This would, theoretically at least, protect the specific antimicrobial such as gepotidacin from selection and/or spread of resistance as well as increase the potency of both compounds. On the other hand, any potential increase in side effects has to be monitored carefully.

In conclusion, with the growing problems of multidrug-resistant *M. genitalium*, an RCT of gepotidacin for the treatment of infections caused by *M. genitalium* is clearly warranted. Combination therapy with doxycycline should also be clinically studied to assess effect and potential protection against development and/or spread of resistance. Finally, a phase III RCT investigating gepotidacin for the treatment of uncomplicated gonorrhoea has recently been initiated, and it would be valuable to investigate eradication of *M. genitalium* as a secondary or exploratory outcome in this RCT.
